# Populations of local direction–selective cells encode global motion patterns generated by self-motion

**DOI:** 10.1126/sciadv.abi7112

**Published:** 2022-01-19

**Authors:** Miriam Henning, Giordano Ramos-Traslosheros, Burak Gür, Marion Silies

**Affiliations:** 1 Institute of Developmental Biology and Neurobiology, Johannes-Gutenberg University Mainz, Mainz 55128, Germany.; 2 Göttingen Graduate School for Neurosciences, Biophysics, and Molecular Biosciences (GGNB) and International Max Planck Research School (IMPRS) for Neurosciences at the University of Göttingen, Göttingen 37077, Germany.

## Abstract

Self-motion generates visual patterns on the eye that are important for navigation. These optic flow patterns are encoded by the population of local direction–selective cells in the mouse retina, whereas in flies, local direction–selective T4/T5 cells are thought to be uniformly tuned. How complex global motion patterns can be computed downstream is unclear. We show that the population of T4/T5 cells in *Drosophila* encodes global motion patterns. Whereas the mouse retina encodes four types of optic flow, the fly visual system encodes six. This matches the larger number of degrees of freedom and the increased complexity of translational and rotational motion patterns during flight. The four uniformly tuned T4/T5 subtypes described previously represent a local subset of the population. Thus, a population code for global motion patterns appears to be a general coding principle of visual systems that matches local motion responses to modes of the animal’s movement.

## Teaser

Local direction–selective cells encode six types of global motion patterns, facilitating the computation of fly self-motion.

## INTRODUCTION

Animals that use the visual system to navigate through their environment need to detect and compute global motion patterns elicited on the eye. These optic flow patterns are generated by locomotion, such as during walking, riding, or flying, where different types of behavior will elicit different optic flow patterns. In the mouse retina, this optic flow generated by self-motion is represented by the population of local motion-sensitive retinal ganglion cells (*1*). Here, directional tuning of retinal ganglion cells changes gradually across visual space, together matching four different types of global motion patterns generated during locomotion: moving forward, retreating, rising, and falling. Thus, the first direction-selective cells in the mammalian visual pathway use a code for visual cues generated by self-motion. Flying animals are exposed to more complex optic flow fields, but how the additional degrees of freedom in behavior affect neuronal processing is not known.

In flies, the first direction-selective cells that encode local motion are the T4 and T5 neurons, which are sensitive to moving ON (T4) or OFF (T5) contrast signals. In contrast to the local direction–selective ganglion cells of the mouse retina, T4/T5 neurons are thought to be uniformly tuned throughout the visual field, representing the four cardinal directions: upward, downward, front-to-back, and back-to-front motions (*2*–*4*). Each of these four directions is thought to be represented by one T4 and one T5 cells in each of the 800 visual units of the fly eye (*5*, *6*). These direction-selective T4/T5 cells compute local motion by comparing inputs from neighboring points in space, represented by neighboring columns in the fly visual system, along one axis (*3*, *4*, *7*–*11*). Neighboring columnar units in the fly visual system are organized in a hexagonal array (*6*), following the hexagonal arrangement of ommatidia in the fly eye. Yet, how these hexagonal interactions can yield four orthogonal motion axes represented by T4/T5 is unknown.

One synapse downstream of T4/T5 cells, optic flow patterns are encoded by wide-field neurons that sample information globally across visual space (*12*–*14*). The general importance of this coding strategy is supported by the widespread presence of such flow field–sensitive cells, covering, for instance, moths (*15*–*17*), locusts (*18*–*20*), and dragonflies (*21*). In blow flies, different wide-field lobula plate tangential cells (LPTCs) are tuned to specific optic flow patterns generated by translational and rotational movements of the animal (*14*, *22*–*25*). LPTCs with a similar receptive-field organization have been mapped in *Drosophila* (*26*–*28*) and are thought to be involved in the control of head optomotor responses, as well as in stabilizing gaze and forward walking (*29*–*31*).

**Fig. 1.**
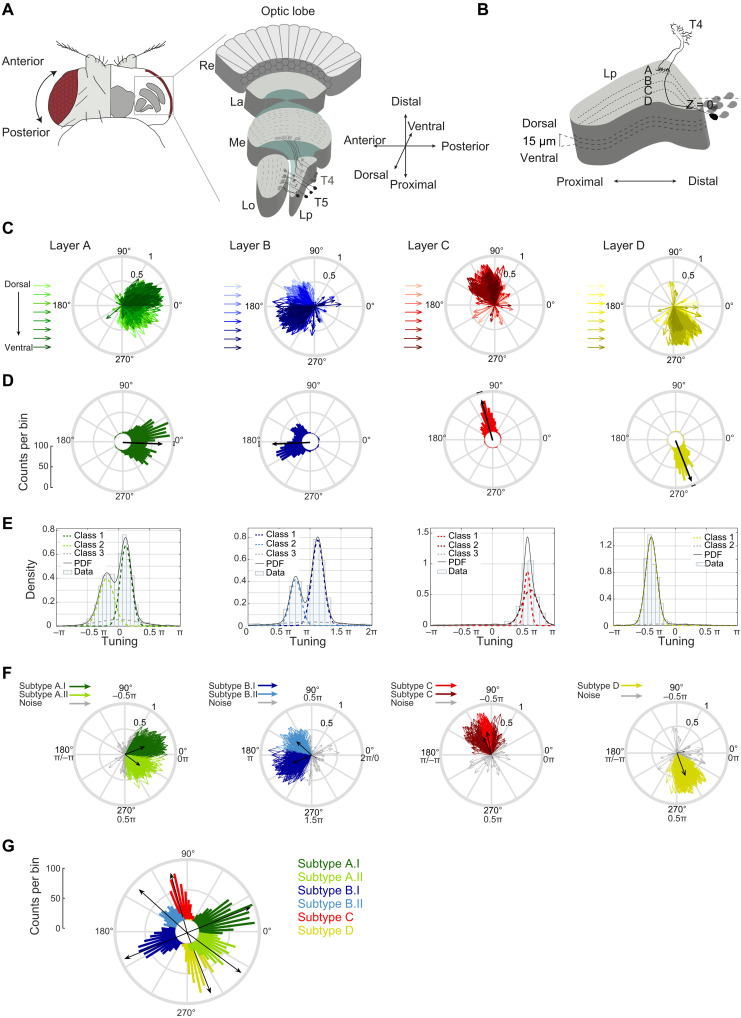
Directional tuning clusters around hexagonal directions of motion. (**A** and **B**) Schematic of the fly visual system and lobula plate. Re, retina; La, lamina; Me, medulla; Lo, lobula; Lp, lobula plate. T4/T5 axon terminals were recorded at different *z*-depths relative to the first T4/T5 cell bodies encountered when moving dorsoventrally (*Z* = 0). (**C**) T4/T5 tuning vectors; lengths depict selectivity (*57*); hue illustrates *z*-depth relative to T4/T5 cell bodies, covering 30, 35, 45, 50, 60, 65, 75, and 80 μm. (**D**) Circular histograms of tuning preference. Black vectors depict average tuning per layer. (**E**) Density distribution of T4/T5 tuning. Solid lines represent the fitted probability density function. Dotted lines show the underlying Gaussian distributions (classes) extracted using the finite Gaussian mixture model SNOB (*36*). In addition to a broad noise distribution, layers A and B contained two main underlying Gaussian distributions with sharp peaks (spread of ~0.5π), and layer D contained one. Two main layer C Gaussian distributions were predicted but were highly overlapping, with peaks separated by less than ~0.1π. When analyzed separately for T4 and T5, only one main class was found (not shown). (**F**) Same as (C), color-coded by class. (**G**) Same as (D), subtypes combined.

To extract optic flow information, wide-field neurons pool information from presynaptic local motion detectors. In *Drosophila*, this is achieved by LPTCs receiving strong input from the columnar T4 and T5 neurons (*2*, *32*). Four T4/T5 subtypes can be distinguished by axonal projections terminating in one of four layers of the lobula plate (Fig. 1A). Here, T4/T5 provide excitatory input to downstream LPTCs within the same layer and indirect inhibitory input to LPTCs of the adjacent lobula plate layer with opposite tuning, thus establishing motion opponency (*32*, *33*). Many LPTCs extend their dendrites along one layer of the lobula plate and thus pool information from one subtype of T4/T5 neurons (*26*, *27*, *34*), although some LPTCs also project to more than one layer (*28*). In addition, local motion signals are selectively amplified within the LPTC dendrites if they match the preferred global motion pattern (*35*). This suggests that the coding of optic flow is fundamentally different between vertebrate and invertebrate visual systems. It is unclear why flies would have evolved a system in which optic flow has to be computed through complex transformations from local motion detectors with uniform tuning to ultimately match the motion patterns generated during flight. To understand how the four subtypes contribute to downstream optic flow fields, it is necessary to have a detailed map of T4/T5 direction tuning across retinotopic space.

Here, we use in vivo two-photon calcium imaging to characterize the direction tuning distribution of T4/T5 neurons across anatomical and visual space. We demonstrate that directional preference of T4/T5 subtypes changes gradually, forming continuous maps of tuning. At the population level, T4/T5 cells are well described by six and not four subgroups that encode six diagonal directions of motion. This matches the number of diagonal directions in the hexagonal lattice of the eye. The six topographic tuning maps match global motion patterns generated by self-motion of the fly. Therefore, the organization of local direction–selective cells that represents global motion patterns parallels the retinal code in the mouse, providing a notable example of convergent evolution. The specific types of optic flow that are encoded differ between the mouse retina and the *Drosophila* visual system, arguing that evolution might have matched neural resources to the different physical distribution of information encountered during walking or flight.

## RESULTS

### T4/T5 population tuning clusters around hexagonal directions of motion

To understand how the T4/T5 neurons contribute to downstream optic flow fields, it is necessary to have a detailed map of T4/T5 direction tuning across retinotopic space. We used in vivo two-photon calcium imaging to record motion responses from large populations of T4/T5 neurons in individual flies. We imaged GCaMP6f in T4/T5 axon terminals in the lobula plate. In each fly, we recorded responses to perspective-corrected visual stimuli, comprising ON and OFF edges moving in eight directions at three different fly orientations relative to the screen. In addition, we obtained signals in each fly in different planes along the dorsoventral axis of the lobula plate. This allowed us to record T4/T5 responses across the dorsofrontal visual field, covering ~100° in azimuth and ~50° in elevation (fig. S1A). To allow comparability of data between flies, we report the anatomical position of the recording relative to T4/T5 cell bodies as an internal reference (Fig. 1B and movie S1). Recordings of 3537 individual units (1376 T4 and 2161 T5), recorded in 14 flies, revealed that tuning of all T4/T5 cells taken together was broad, together spanning 360° of motion (Fig. 1C). When just looking at layer A neurons, individual neurons were not all tuned to front-to-back motion but spanned directional tuning ranging from 60° to −60° (300°). Overall, neurons in both layers A and B covered more than 120° of tuning direction, whereas cells of layers C and D were tuned to a range of ~60° (Fig. 1C). Thus, within the recorded volume of the lobula plate, cells of layers A and B covered twice the directional tuning range of cells in layers C and D. Furthermore, dorsoventral location appeared to strongly affect tuning direction in layers A and B (Fig. 1C). In layer A, cells that were more dorsally located in the lobula plate preferentially covered the 300° to 360° range, whereas more ventral cells of the lobula plate showed tuning directions in the 0° to 60° range. In layer B, more dorsally located cells were tuned to the 120° to 180° range, and more ventrally located cells were tuned to 180° to 240° (Fig. 1C). Although the population of T4/T5 cells covered all directions of motion, the tuning distribution was nonuniform (circular Rayleigh test, *P* < 0.0001).

Looking at the number of neurons sensitive to a certain motion direction, most neurons in layers A and B were tuned to the diagonal directions of motion (Fig. 1D). This indicates that individual tuning differs from the overall average orthogonal tuning of these layers, i.e., from front-to-back or 0° tuning for layer A and from back-to-front or 180° tuning for layer B described previously (*2*–*4*). Cells in layers C and D each showed a unimodal directional tuning distribution in the upward or downward direction, respectively (Fig. 1D), which was well fit by one Gaussian (layer C: *R*^2^ = 0.96, layer D: *R*^2^ = 0.96). In comparison, fits by one Gaussian for layers A and B were worse (layer A: *R*^2^ = 0.86, layer B: *R*^2^ = 0.76), and these layers were fit better by a sum of two Gaussians (layer A: *R*^2^ = 0.99, layer B: *R*^2^ = 0.99). In layers A and B, an unsupervised statistical model based on the minimum message length principle [SNOB; (*36*)] predicted a bimodal distribution in layers A and B, well fit by two Gaussians, as well as one underlying noise distribution (Fig. 1E). When assigning each cell to one of six classes based on this SNOB analysis, tuning of two subtypes in layer A or B split at 0° or 180°, respectively (Fig. 1F). The population average of the A.I class was tuned to diagonal upward motion (~30°), and the A.II class was tuned to diagonal downward motion (~330°), separating the two by 60°. Layer B classes encoded the two opposite axes of motion direction (Fig. 1F). Average motion tuning within individual classes reveals sensitivity to six directions (Fig. 1F), matching the number of diagonals in the hexagonal arrangement of the fly compound eye (Fig. 1G and fig. S1B).

**Fig. 2.**
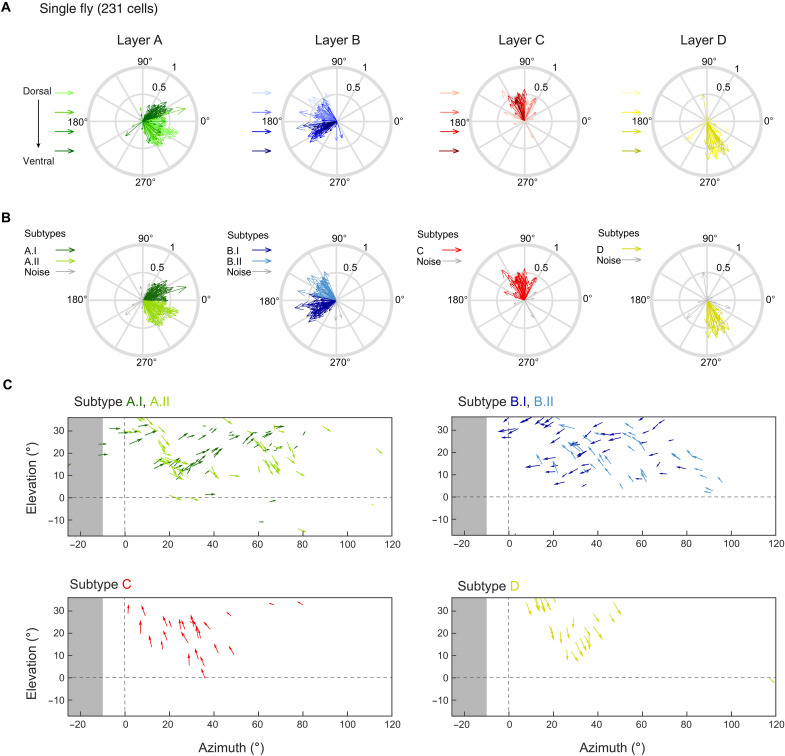
Six T4/T5 subtypes covering overlapping regions in visual space exist in one fly. (**A**) Compass plots showing directional tuning of individual neurons in one fly. Motion responses were represented by a vector whose direction depicts tuning, whereas its length indicates selectivity (*57*). Hue illustrates *z*-depth relative to a reference (the outermost T4/T5 cell bodies), covering 30, 45, 60, and 75 μm from light to dark. (**B**) Same as (A) but tuning of individual neurons is color-coded on the basis of class identity. (**C**) Individual T4/T5 tuning plotted at their receptive field center coordinates in visual space. Arrows depict tuning direction, and vector length indicates direction selectivity. Same data from one fly as in (A) and (B), without cells belonging to the noise distributions. Each plot shows data from one anatomical layer, color-coded by class identity from the SNOB model. Horizontal and vertical dashed lines mark the split between left and right visual hemispheres and the horizon, respectively. Gray shaded areas indicate the visual space in the left hemisphere that cannot be seen by the right eye.

The same distributed tuning of T4/T5 neurons was found within individual flies: Neurons that were more dorsally located in the lobula plate appeared to be tuned to diagonal downward motion in the front-to-back direction in layer A and diagonal upward motion in the back-to-front direction in layer B (Fig. 2A). The more ventrally located cells appeared to represent the other diagonal. Neurons of both layer A and B classes, as identified by the SNOB analysis, were found within one fly and each class spanned a directional tuning range of ~60° (Fig. 2B). We then asked how the individual classes with their different tuning preferences were represented in visual space. To do so, we mapped the receptive fields of all cells recorded within one fly, linking anatomical position with visual space (fig. S2). Plotting the tuning direction of each neuron back onto visual space showed that the six different classes responded to largely overlapping regions of visual space (Fig. 2C). Neurons of layer A whose receptive fields covered similar regions of visual space showed very different tuning when the two neurons were coming from two different classes. Instead, nearby neurons from one class were similarly tuned. Within layers C and D, neurons with neighboring receptive fields always were similarly tuned to upward (layer C) or downward (layer D) motion across visual space. Together, these data argue that visual space is represented by six different types of T4/T5 neurons: two layer A and two layer B types, as well as one type for layers C and D. At the population level, the six functional T4/T5 subtypes cover all tuning directions.

**Fig. 3.**
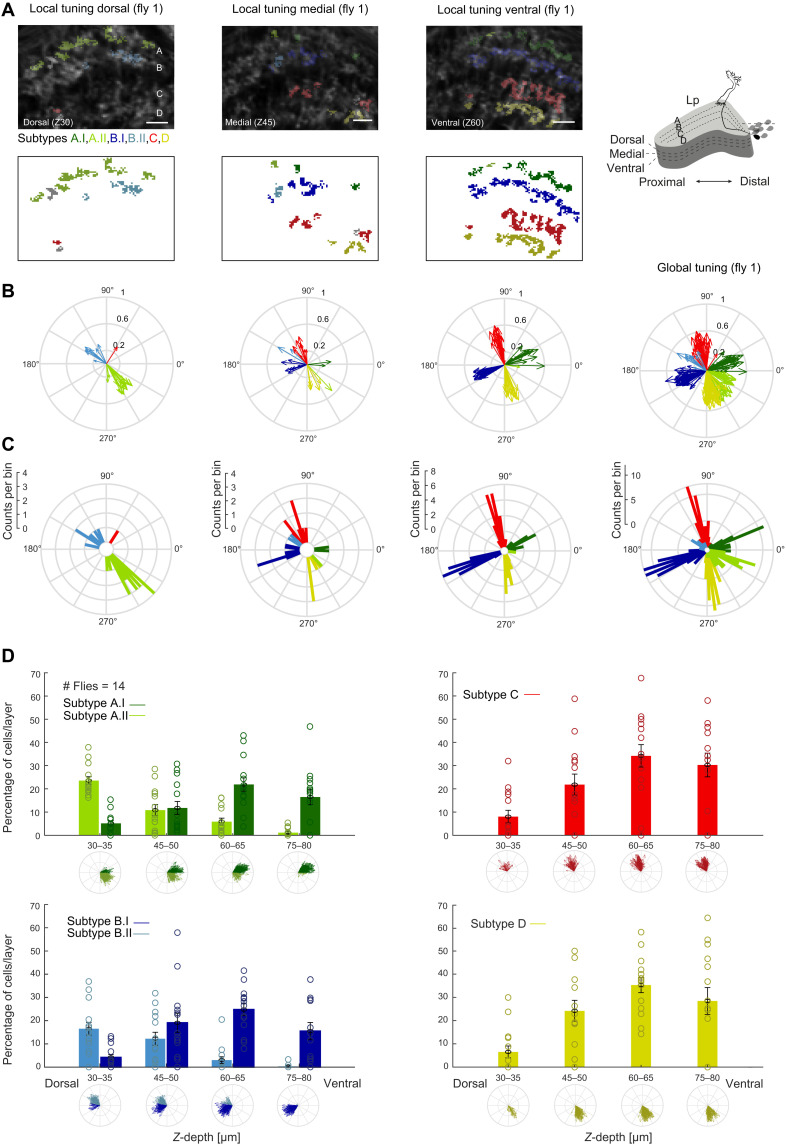
Layer A and B subtype projections separate along the dorsoventral axis of the lobula plate. (**A**) Top: In vivo two-photon calcium images of the lobula plate at three planes along the dorsoventral axis (z30/z45/z60 = *z*-depth 30/45/60 μm). ROIs are color-coded on the basis of their subtype identity. Bottom: Same data shown without background lobula plate image. Scale bars, 10 μm. (**B**) Tuning of individual neurons from one fly recorded at different planes along the dorsoventral axis of the lobula plate [same data as in (A)], as well as global tuning within this fly. (**C**) Same data as in (B), shown as circular histograms. (**D**) Histograms displaying percentage of neurons within one lobula plate layer found at a certain *z*-depth in the lobula plate, separated by subtype identity for layers A and B, such that data in each of the four panels add up to 100%. Bars show means ± SEM. Individual percentages are shown for each fly (*N* = 14).

To understand the spatial organization of six T4/T5 subtypes projecting to four anatomically distinguishable lobula plate layers, we plotted cellular subtype identity back onto the anatomical structure of the lobula plate (Fig. 3A and fig. S3). One lobula plate layer recorded in one plane along the dorsoventral axis predominantly housed one of the two layer A and one of the two layer B subtypes. At more ventral planes, subtypes A.I and B.I as well as the single respective subtypes of layers C and D were found more frequently. Dorsal planes more prominently housed subtypes A.II and B.II but hardly showed any layer C or D cell responses (Fig. 3, A to C, and fig. S3). Thus, most local T4/T5 recordings in an individual fly preferentially showed either four subtypes [as, e.g., described in (*2*, *3*)] or two subtypes, each representing snapshots of the T4/T5 population. Only intermediate planes showed both subtypes, separated along the proximodistal axis (Fig. 3, A to C). This separation of subtypes along the dorsoventral axis is also seen when analyzed across all flies (Fig. 3D). This argues for a spatial separation of layer A and B subtypes at the level of T4/T5 axon terminals. Only a global analysis of tuning revealed the six T4/T5 subtypes encoding six diagonal directions of motion (Fig. 3, B and C).

### T4/T5 neurons form topographic maps of directional tuning

**Fig. 4.**
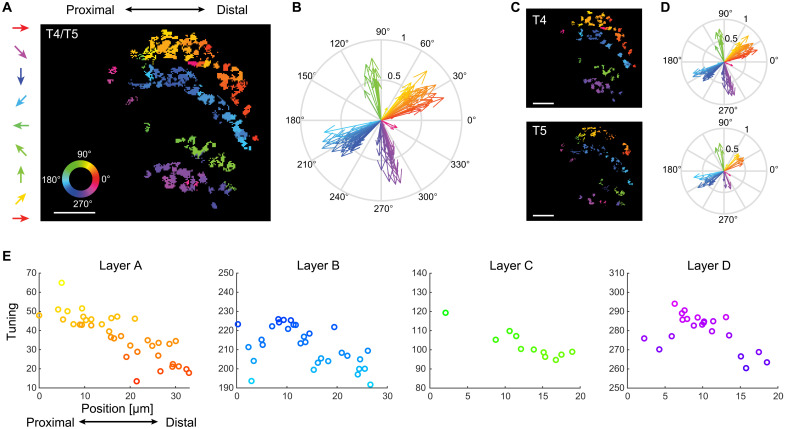
T4/T5 neurons of one subtype form topographic maps of directional tuning in the lobula plate. (**A**) Image of one layer of the lobula plate with ROIs color-coded according to their directional tuning. Note that this is a different color code as compared to the one highlighting subtypes used in previous figures. (**B**) Data from (A) shown in vector space. (**C** and **D**) Same data as in (A) and (B), shown separately for T4 and T5 neurons. Scale bars, 10 μm. (**E**) Directional tuning as a function of anatomical position along the proximodistal axis within one lobula plate layer. Data from a range of 60° of tuning are shown, limiting this illustration to subtypes A.I and B.I.

We next asked how the tuning of individual neurons within the ~60° distribution covered by one subtype relates to the anatomical organization of the lobula plate. We reasoned that the distributed directional tuning within one subtype could be noise or could reflect a topographical organization of tuning direction. Color-coding axon terminals based on their directional preference revealed that the tuning of neighboring cells was similar and gradually changed along the distal-to-proximal axis (Fig. 4). As such, recording in one ventral plane of layer A (subtype A.I) revealed T4/T5 tuning ranging from diagonally upward on the proximal end to front-to-back motion on the distal end of the lobula plate (Fig. 4, A and B). T4/T5 cells of other subtypes also gradually changed tuning from proximal to distal (Fig. 4, A, B, and E). Subtler changes in the tuning of neighboring cells within one subtype were also apparent along the dorsoventral axis but showed abrupt changes in tuning at the subtype boundary (fig. S4, A and B). This gradually distributed tuning existed for both T4 and T5 when analyzed separately (Fig. 4, C and D, and fig. S4C). Because T4/T5 neurons are retinotopically organized, this directional tuning map suggests that the population of T4/T5 cells is sensitive to specific global motion patterns.

### The six T4/T5 subtypes encode optic flow induced by self-motion

**Fig. 5.**
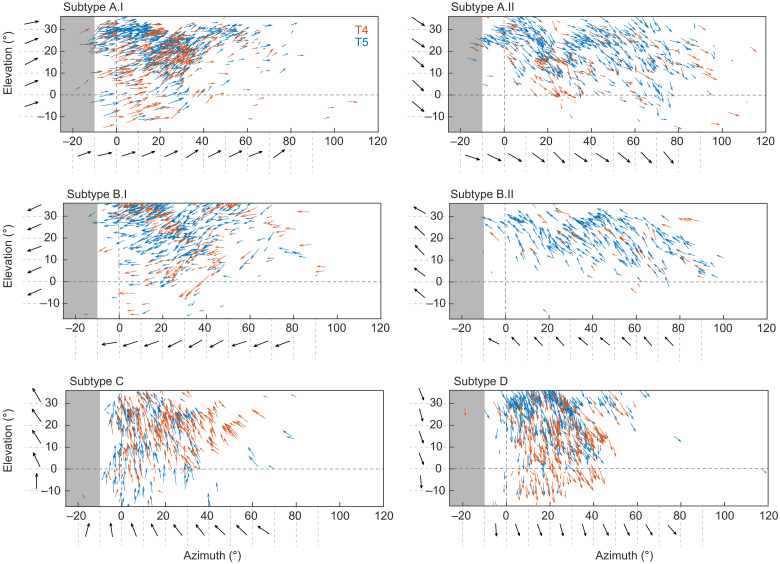
Population of T4/T5 neurons encodes global motion patterns. Arrows indicate tuning direction of individual neurons plotted at their receptive field center coordinates in visual space. Length of vectors indicates direction selectivity of T4 (red) or T5 neurons (blue) (*n* = 14 flies). Horizontal and vertical dashed lines mark the split between left and right visual hemispheres and the horizon, respectively. Black tuning vectors show mean across 10 degree-wide bins. Gray shaded areas indicate the visual space in the left hemisphere that cannot be seen by the right eye.

The local differences in the tuning preference within one T4/T5 subtype are reminiscent of direction-selective ganglion cells in the vertebrate retina, where the population of cells encodes translational optic flow generated by self-motion of the animal (*1*). We hypothesized that the differential tuning measured within each subtype of T4/T5 cells in the fly visual system serves a similar function. To relate direction tuning to the visual input, we mapped receptive-field centers of all cells imaged across 14 flies and plotted tuning at each receptive-field location on the screen (as done for one fly in Fig. 2C and fig. S2). This revealed that, in visual space, cells of one subtype do not encode a uniform direction of motion, but rather that direction tuning of all cells within one subtype changes gradually across visual space (Fig. 5 and fig. S5, A and B). This was both true for T4 and T5 (Fig. 5). These topographic tuning maps resemble global motion patterns generated by different directions of self-motion in the fly. Global directional tuning patterns were similar for the individual flies, and within a small region of visual space, neurons from different flies shared the same directional tuning (fig. S5C), arguing for little fly-to-fly variation. Across the different classes, T4/T5 neurons in layers C and D appear to encode optic flow generated by downward or upward movement of the fly, whereas T4/T5 neurons in layers A and B seem to be tuned to diagonally upward or downward motion. The two flow fields encoded by the two subtypes of layers A or B are vertically flipped versions of each other (Fig. 5). The successive change of tuning along azimuth and elevation matches the change of tuning seen in the topographic maps in the lobula plate (Figs. 4 and 5 and fig. S5).

**Fig. 6.**
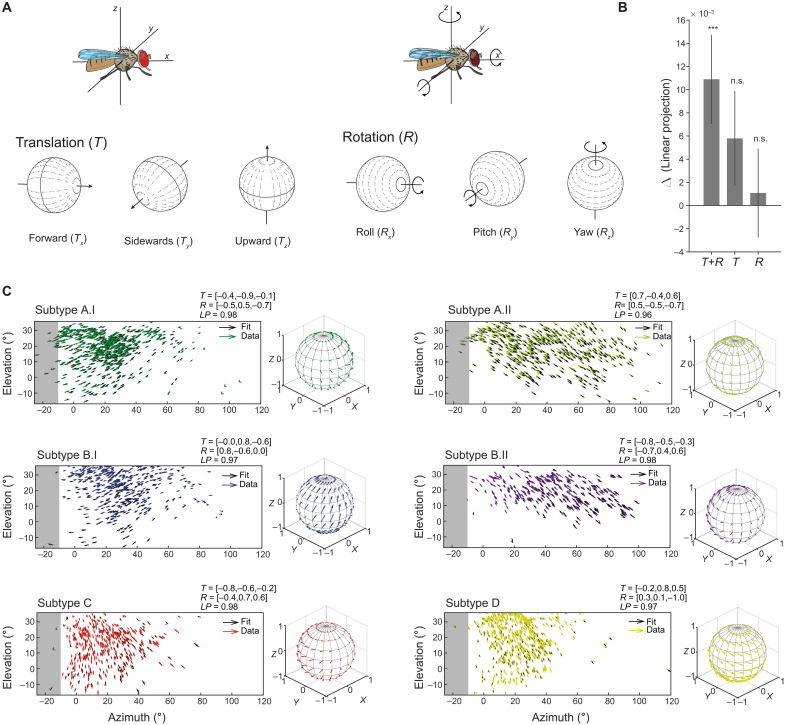
T4/T5 global motion patterns are well fit by motion patterns generated by self-motion. (**A**) Schematic of rotational and translational flow fields around the three body axes of the fly [reused with permission from Springer Nature Customer Service Centre GmbH: (*60*)]. (**B**) Differences of fit quality for three model types [translation + rotation (*T*+*R*), pure translation (*T*), pure rotation (*R*)] compared to a uniform vector field model (Wilcoxon, ***P < 0.001). Bars show means ± SD across 10-fold cross-validations for all six subtypes. (**C**) Flow fields of data from all subtypes and the fitted normalized T+R optic flow model. Model vectors (black) are shown for each corresponding data vector. The model fit is shown on a 3D sphere to the right of each panel. n.s., not significant.

To investigate the type of self-motion encoded by the different subtypes, we trained an optic flow model (*37*) to match the population receptive fields of T4/T5 neurons. We fitted the parameters for the three axes of motion for both translation (*T_x_*, *T_y_*, and *T_z_*) and rotation (*R_x_*, *R_y_*, and *R_z_*) for a fixed distance of the visual space to the fly (Fig. 6A). Although the population data did not fully cover the visual field of one eye, 10-fold cross validation of the model fits produced similar global tuning patterns across the whole visual field (fig. S6A). Optic flow fields generated by the model were well-matched filters for the global tuning patterns of each of the six T4/T5 subtypes (Fig. 6, B and C). We compared performance including models where the fly only turned (rotational optic flow) or moved straight (translational optic flow). Across the six subtypes, the model combining rotations and translations outperformed the null model consisting of a uniform vector field, with larger performance in layers A.I, B.II, C, and D (Fig. 6B and fig. S6B). Thus, T4/T5 subtypes are tuned to optic flow generated by complex mixtures of translational and rotational motion (Fig. 6C). The translational components are reflected in the global motion pattern that were obtained from those fits, which show either points of convergence or expansion within the visual field of the eye (fig. S6A). These points generally do not lie along the horizon or along the vertical axis straight ahead, but either at more positive or negative degrees of azimuth and elevation, consistent with the observation that the global motion patterns represent diagonal motion directions (fig. S6A). This finding is also present in the maps of LPTCs (*24*, *25*). Together, our data show that local direction–selective T4/T5 neurons display a population code for global motion patterns, which might then facilitate the downstream computation of different types of flow fields elicited by self-motion in LPTCs. Populations of T4/T5 cells are thus tuned to optic flow patterns, similar to their vertebrate counterparts (*1*) but representing six instead of four global motion patterns.

## DISCUSSION

In this study, we have demonstrated that optic flow is encoded by the local motion detectors in the *Drosophila* visual system: The direction-selective T4/T5 neurons are divided into six subtypes that encode specific optic flow patterns. Within each of the six subtypes, individual tuning preference is gradually distributed across the lobula plate such that the population of T4/T5 neurons of each subtype together forms a retinotopic tuning map that encodes global motion patterns containing information about translational and rotational self-motion of the fly.

Direction-selective T4/T5 neurons in *Drosophila* have been described to encode four cardinal directions of motion (*2*, *3*). Population T4/T5 recordings now reveal average tuning to diagonal rather than cardinal motion directions such that six subtypes of T4/T5 neurons exist. Only a global analysis of directional tuning reveals these six subtypes, but tuning to diagonal motion has been observed in electrophysiological recordings of an individual T4 neuron (*10*) and in optical recordings of T4/T5 (*3*, *38*). T4/T5 neurons compute direction-selective signals across neighboring columns within the eye (*5*, *7*). Thus, motion can mainly be computed along the internal organization of the fly eye, and the hexagonal arrangement of the eye does not need to be transformed into a cardinal coordinate system.

Individual directional preference of a T4/T5 neuron correlates with its dendrite orientation, which manifests during development (*6*, *39*, *40*), such that dendrites of layer C T4/T5 neurons, for example, point down (ventrally), whereas layer D T4/T5 dendrites point up (dorsally) (*40*). Developmental dendrites of subtypes A and B instead predominantly project along the diagonal (*40*), i.e., in the dorso-anterior and ventro-anterior direction for layer A neurons and in the dorso-posterior and ventro-posterior direction of layer B neurons. This developmental dendrite orientation is thus consistent with their subsequent adult distribution of direction selectivity. Our data show that the six T4/T5 subtypes retinotopically cover overlapping regions in visual space. This seemingly contradicts the notion that there are four T4/T5 subtypes per column (*2*, *6*). One possibility is that each column houses one of the two layer A and B subtypes, respectively, decreasing spatial acuity. However, electron microscopy (EM) reconstructions found 60 T4s in a dataset reconstructing seven columns (*6*). This is the number one would expect if each of the seven columns housed six T4 cells plus the neighboring T4 cells that extend their dendrites from the outside, given that each T4 neuron extends its dendrites across three neighboring columns (*6*, *7*). A full connectome and a comprehensive analysis of T4/T5 dendrite anatomy across the visual system will be needed to clarify how adult dendrite orientation is distributed across the visual system to represent the six subtypes. Furthermore, single-cell transcriptomics has assigned developing T4/T5 cells to distinct clusters based on their genetic profiles (*40*–*43*), but genes involved in dendrite development or the differentiation are expressed in narrow time windows (*43*). One recent study identified a genetic subpopulation of T4 neurons, restricted to lobula plate layers A and B (*41*). It remains to be determined whether this corresponds to the functional layer A/B subtypes, and genetic access will help to better understand the development and anatomy of the individual subtypes.

While downstream of T4/T5, wide-field LPTCs are thought to encode self-motion (*22*, *27*), our data show that the population of T4/T5 cells already encodes optic flow generated by a combination of rotational and translational self-motion of the fly. Within an optic flow field, single T4/T5 tuning changes along the retinotopic map. This could be inherited by the spatial distribution of ommatidia along the optical axis, which varies with the curvature of the eye (*44*, *45*). T4/T5 can then pass this information to downstream LPTCs. Comparing the global motion patterns encoded by T4/T5 with the motion patterns encoded by certain LPTCs shows both interesting similarities and differences. For example, the *Drosophila* LPTC horizontal system north (HSN) extends its dendrites solely in layer A of the lobula plate, and its receptive field resembles the subtype A.I global motion pattern [Figs. 5 and 6 and (*27*)]. However, the broader receptive field of the well-characterized HSN in blowflies appears to combine two components of diagonal upward and diagonal downward motion, suggesting that this optic flow field could originate by combining T4/T5 subtype A.I and A.II information. Close to 20 LPTCs have been characterized in *Drosophila* (*28*, *46*–*48*), and more are likely to exist given strong similarities with blow flies, with its 60 LPTCs (*23*, *48*). Overall, it appears as if the LPTCs do not need to transform cardinal motion information into complex flow fields but that these can instead be composed of T4/T5 global motion patterns. This facilitates the encoding of self-motion by various types of LPTCs (*14*, *22*–*24*, *26*–*28*). Further internal dendritic processing, such as suppression of adjacent local motion signals, electrical coupling between LPTCs (*27*), and feedforward inhibition from lobula plate intrinsic neurons (*33*), will support the computation of diverse optic flow fields (*32*, *33*, *35*).

The encoding of optic flow generated by self-motion at the level of local motion detectors has also recently been described in the mouse retina (*1*), where any kind of self-motion will activate different retinal ganglion cell types from both eyes in a unique pattern that will be decomposed into translational and rotational components further downstream. The fly eye and the vertebrate retina both show differences between local and global directional tuning (*1*, *49*), and similarly compute visual signals generated by self-motion at the population level (*1*). A population code for optic flow generated by self-motion might therefore be a canonical strategy of visual systems and evolved convergently during evolution. However, mice and flies differ in the number and directions of optic flow encoded by local direction–selective cells. Flying animals encode more motion axes than walking animals, likely to match the higher degrees of freedom encountered during flight. This difference might highlight adaptation to the visuoecological niches of flying and walking animals. We are just starting to understand how a population code in visual systems matches the statistics of the visual environment (*1*, *50*–*53*) or animal behavior. Thus, this work is an important step toward understanding how anatomy, ethological constraints, and neuronal function are ultimately linked.

## MATERIALS AND METHODS

### *Drosophila* strains and fly husbandry

*Drosophila melanogaster* were raised on molasses-based food at 25°C and 55% humidity in a 12-hour light/12-hour dark cycle. For all imaging experiments, female flies of the genotype *w^+^; R59E08-LexA^attP40^, lexAop-GCaMP6f-p10^su(Hw)attp5^/R59E08-LexA^attP40^, lexAop-GCaMP6f-p10^su(Hw)attp5^* were recorded 3 to 5 days after eclosion at room temperature (20°C). *R59E08-LexA^attP40^* and *lexAop-GCaMP6f-p10^su(Hw)attp5^* were obtained from the Bloomington Drosophila Stock Center (BDSC; #52832 and #44277), recombined, and crossed into a *w^+^* background. The *R59E08-LexA^attP40^* line shows full coverage of T4/T5 neurons, as tested by full overlap with the expression pattern of the commonly used *R42F06-Gal4^attP2^* T4/T5 driver line (*2*, *3*).

### In vivo two-photon calcium imaging

#### Fly preparation, experimental setup, and data acquisition

Before two-photon imaging, flies were anesthetized on ice and fit into a small hole in stainless-steel foil that was designed to match the size of the fly head and thorax, located in a custom-made holder. The head was tilted approximately 30° to expose the back of the head. To fix the head of the fly, a small drop of ultraviolet-sensitive glue (Bondic) was used on the left side of the brain and the thorax. Using this mounting method, head angle between flies differed by ~0.5°, measured by the alignment of the pseudopupil between the two eyes (in *n* = 10 flies). The cuticle on the right eye, fat bodies, and tracheae were removed using breakable razor blades and forceps. To ensure constant nutrients and calcium supply, flies were perfused with a carboxygenated saline containing 103 mM NaCl, 3 mM KCl, 5 mM *N*-tris(hydroxymethyl)methyl-2-aminoethanesulfonic acid (TES), 1 mM NaH_2_PO_4_, 4 mM MgCl_2_, 1.5 mM CaCl_2_, 10 mM trehalose, 10 mM glucose, 7 mM sucrose, and 26 mM NaHCO_3_ (pH 7.3). To record calcium activity, a two-photon microscope (Bruker Investigator, Bruker, Madison, WI, USA), equipped with a 25×/1.1 objective (Nikon, Minato, Japan), was used. For excitation of GCaMP6f, the excitation laser (Spectra-Physics InSight DS+) was tuned to a wavelength of 920 nm with <20 mW of laser power measured at the objective. Emitted light was filtered through an SP680 short-pass filter, a 560 lpxr dichroic filter, and a 525/70 emission filter and detected by photomultiplier tubes set to a gain of 855 V. Imaging frames were acquired at a frame rate of ~15 to 20 Hz and 5× optical zoom, corresponding to a pixel size of ~0.5 μm using PrairieView software. Each fly was recorded in at least three to five different focal planes (*z*-depth). We determined *z*-depth position relative to cell bodies and started the first recording at a z-depth of 30 to 35 μm from there (movie S1). A total of 3537 cells in 14 flies, with 479/926 layer A, 252/662 layer B, 365/220 layer C, and 280/353 layer D T4/T5 cells, were recorded. Planes were then imaged every 15 μm (Fig. 1B).

#### Visual stimulation

Visual stimuli were presented on a 9 cm–by–9 cm rear projection screen in front of the fly covering a visual angle of ~80° in azimuth and ~55° in elevation. To cover a larger part of the horizontal visual field of ~168°, we rotated the fly with respect to the screen two times by 45° and recorded each fly at three positions relative to the screen (fig. S1A). In total, we thus stimulated an area of the visual field ranging from −34° to 134° in azimuth and −17° to 36° at the closest point of the screen to the fly in elevation (fig. S1A). Note that results are just plotted in a range between −23° and 120° in azimuth, as no neuronal responses were measured to the stimulus beyond that visual area. Stimuli were filtered through a 482/18 bandpass filter (Semrock) and ND1.0 neutral density filter (Thorlabs) and projected using a LightCrafter 4500 DLP (Texas Instruments, Texas, USA) with a frame rate of 100 Hz and synchronized with the recording of the microscope as described previously (*54*). Visual stimuli were generated using custom-written software using C++ and OpenGL. To correct for distortions due to the fly’s viewing position relative to the screen, stimuli were drawn on a virtual cylindrical surface and perspective-corrected using frustum.

#### Moving OFF and ON edges

Full-contrast dark or bright edges moved with a velocity of 20°/s across the full screen to four or eight different directions. Each stimulus direction was presented at least twice in pseudo-random order. The four-direction stimulus was merely used for the subsequent identification of T4 and T5 axon terminals.

### Data analysis

#### Preprocessing

All data analysis was performed using MATLAB R2017a (The MathWorks Inc., Natick, MA) or Python 2.7. Motion artifacts were corrected using Sequential Image Alignment SIMA, applying an extended hidden Markov model (*55*).

#### Automated ROI selection

For the extraction of single T4 or T5 axon terminals, we made use of their contrast- and direction-selective responses to ON and OFF edges moving into four directions. First, the aligned images were averaged across time, and the average image intensity was Gaussian-filtered (*s* = 1.5) and then threshold-selected by Otsu’s method (*56*) to find foreground pixels suitable for further analysis. After averaging responses across stimulus repetitions, we selected pixels that showed a peak response larger than the average response plus two times the SD of the full trace. These pixels were grouped on the basis of their contrast preference (ON or OFF pixels) and further assigned to four categories based on their anatomical location within the lobula plate (layer A, B, C, or D). We further calculated a direction-selectivity index (*DSI*) and contrast selectivity index (*CSI*) for each pixel as follows

$$\mathrm{DSI}=\frac{{\mathrm{PD}}_{\text{max}}-{\mathrm{ND}}_{\text{max}}}{{\mathrm{PD}}_{\text{max}}}$$

$$\mathrm{CSI}=\frac{{\mathrm{PC}}_{\text{max}}-{\mathrm{NC}}_{\text{max}}}{{\mathrm{PC}}_{\text{max}}}$$

where *PD*_max_ and *ND*_max_ denote the maximal response into the preferred direction (*PD*) and null direction (*ND*) and *PC*_max_ and *NC*_max_ denote the maximum responses for the preferred contrast (*PC*) and the nonpreferred or inverse contrast (*NC*). We excluded all pixels that did not exceed the *CSI* threshold of 0.2 to obtain clean T4 or T5 responses. For the final clustering, we used the quantified *DSI* and *CSI* parameters and the timing of the response to the *PD*. On the basis of these parameters, the Euclidean distance between each pair of pixels was calculated and average-linkage agglomerative hierarchical clustering was performed. We further evaluated the optimal distance threshold that yielded most clusters of the appropriate size between 1.25 and 6.25 μm^2^. All resulting clusters that fell outside this range were excluded from further analysis. Cluster locations were saved and matched with subsequent recordings of the same cells to other stimulus types.

#### Moving OFF and ON stripes

For *dF*/*F* calculation, baseline responses to ~0.5-s gray epoch were used. To quantify direction selectivity of single cells, responses were trial-averaged and the peak response to the eight different directions of either increment or decrement bars was extracted for T4 and T5 cells, respectively. We further quantified the tuning of single cells by computing vector spaces as follows (*57*)

$$L_{\text{dir}}=|\frac{\sum_{k}R(\theta_{k})\text{exp}(i\theta_{k})}{\sum_{k}R(\theta_{k})}|$$

where *R*(θ*_k_*) is the response to angle θ*_k_*. The direction of the vector *L*_dir_ denotes the tuning angle of the cell, and the normalized length of the vector is related to the circular variance and thus represents the selectivity of the cell.

#### Receptive-field center extraction

To extract receptive-field centers, we used a back-propagation algorithm to map the receptive fields of T4 and T5 cells and to locate the center of the receptive fields (*58*). First, we imaged neural responses to eight different directions and created two-dimensional (2D) images from 1D response traces. Neural latency and indicator dynamics introduce delays that will decrease the precision of receptive-field position estimation. To account for this delay, we measured the spatial difference of the response peaks between a static and a moving stimulus. We found an average of 9.6° delay for both T4 and T5 cells and shifted the traces for 9.6° before calculating the receptive-field map in our back-propagation algorithm (fig. S2, A and B). These were rotated according to their corresponding direction and averaged to obtain a receptive-field map. To find the center of the receptive field, we fitted a 2D Gaussian and took its peak coordinate.

#### *Z*-stack generation

Images representing the location of single region of interest (ROI) color-coded by their directional preference were generated in MATLAB. Images containing data from different *z*-depth layers within the same fly were then further processed in Illustrator to create pseudo *z*-stacks. For this, ROIs from the same lobula plate layer were first compiled in a 3D structure, and ROIs from different *z*-depth layers were stacked to better represent the third dimension of the lobula plate.

### Statistics

All statistics were done in MATLAB using Circular Statistics Toolbox (*59*).

#### SNOB analysis

To extract underlying classes from the population of neurons found in layers A and B, we converted data of each population to be linear in the range of directions that most neurons where selective to, resulting in a scale from −π to π for layers A, C, and D and a scale from 0 to 2π for the data from layer B. We used the finite mixture model SNOB (*36*) to predict the number of underlying Gaussians using minimum message length criterion. We further used the statistical prediction from the model to assign individual neurons to each of the underlying classes by choosing the class with the highest probability of the neuron’s tuning preference (Fig. 2, E and F).

### Model

We fitted an optic flow field elicited from self-motion on the field of view at a constant distance from the observer, i.e., a spherical surface. Two coordinates describe the viewing direction: the azimuth θ and the elevation ϕ angles.

The self-motion flow-field vectors ${\vec{p}}_{i}$ at each viewing location ${\vec{d}}_{i}$ on the unit sphere were specified by the translation and rotation vectors, ${\vec{v}}_{T}=(v_{\mathrm{Tx}},v_{\mathrm{Ty}},v_{\mathrm{Tz}})$ and ${\vec{v}}_{R}=(v_{\mathrm{Rx}},v_{\mathrm{Ry}},v_{\mathrm{Rz}})$, respectively (*37*)

$$\vec{p_{i}}=-({\vec{v}}_{T}-({\vec{v}}_{T}\cdot {\vec{d}}_{i}){\vec{d}}_{i})-{\vec{v}}_{R}\times {\vec{d}}_{i}$$

The flow-field vectors were then represented in spherical coordinates ${\vec{p}}_{i}=u_{i}{\hat{e}}_{\theta }+v_{i}{\hat{e}}_{\phi }+r{\hat{e}}_{r}$ to extract a vector tangential to the spherical surface $\vec{q_{i}}=(u_{i},v_{i})$ that could be matched to the direction-selectivity vectors from T4/T5 data. The uniform flow-field tangent to the spherical surface was specified by a single vector ${\vec{v}}_{U}=\vec{q_{i}}=(u,v)$ at every viewing position.

The comparison of data to model was done using the following loss function

$$\begin{array}{cc} ℒ(\{{\vec{q}}_{i,\text{data}},{\vec{q}}_{i,\text{model}}|i\in [1,N]\}) & =\frac{\sum_{i=1}^{N}{\vec{q}}_{i,\text{model}}\cdot {\vec{q}}_{i,\text{data}}}{\sum_{i=1}^{N}\|{\vec{q}}_{i,\text{model}}\|\cdot \|{\vec{q}}_{i,\text{data}}\|}\\ & =\frac{\sum_{i=1}^{N}\|{\vec{q}}_{i,\text{model}}\|\cdot \|{\vec{q}}_{i,\text{data}}\|\text{cos}\eta_{i}}{\sum_{i=1}^{N}\|{\vec{q}}_{i,\text{model}}\|\cdot \|{\vec{q}}_{i,\text{data}}\|} \end{array}$$

where η*_i_* is the angle between the model and the data flow vectors at location ${\vec{d}}_{i}$, for all *N* vectors in the dataset, and ‖ · ‖ indicates the magnitude of the vector. When all vectors match in both magnitude and direction, this quantity is 1, and when all vectors match in magnitude but are in opposite directions, this quantity is −1. To optimize for the vectors ${\vec{v}}_{T}=(v_{\mathrm{Tx}},v_{\mathrm{Ty}},v_{\mathrm{Tz}})$ and ${\vec{v}}_{R}=(v_{\mathrm{Rx}},v_{\mathrm{Ry}},v_{\mathrm{Rz}})$, and ${\vec{v}}_{U}=\vec{q_{i}}=(u,v)$ that maximize ℒ, the MATLAB function fmincon was used. The positive of the loss function is the linear projection (LP), shown in Fig. 6 (B and C) and fig. S6B.

Four model variations were considered: fitting both ${\vec{v}}_{R}$ and ${\vec{v}}_{T}$, fitting ${\vec{v}}_{R}$ with ${\vec{v}}_{T}=\vec{0}$, ${\vec{v}}_{T}$ with ${\vec{v}}_{R}=\vec{0}$, and fitting the uniform model ${\vec{v}}_{U}$. For all cases, the model was constrained to vectors of unit magnitude, to focus on the direction rather than the speed of self-motion, and because the T4/T5 vectors (DSI) had magnitudes between 0 and 1.

The data were fitted using 10-fold cross-validation (CV), dividing the data into 10 random subsets. In each fold, nine subsets were used for training and the remaining subset was used for testing the model fit. For each CV fold, the same training data were fit 10 times starting from 10 different random conditions, and the best fit was stored and used to calculate the performance on the test set. The same training and testing data were used for all models, resulting in repeated measures of the test performance across models. Statistical testing was done on the 10 test-performance values obtained per model. A one-tailed nonparametric Wilcoxon signed-rank test was used to determine whether the performance of each of the self-motion models was higher than the performance of the uniform model, for tests pooling all subtypes (Fig. 6B) and tests of individual subtypes (fig. S6B). A signed-rank test accounted for repeated measures, and a Bonferroni correction was applied to account for multiple testing (*P* < 0.05/3). 

## Notes

View/request a protocol for this paper from *Bio-protocol*.
