# Supplementary material for: Populations of local direction–selective cells encode global motion patterns generated by self-motion
Source: Sci Adv. 2022 Jan 19;8(31):eabi7112. doi: 10.1126/sciadv.abi7112 (PMC8769539; doi:10.1126/sciadv.abi7112)
Supplement: Supplementary file 1 — Figs. S1 to S6 Legend for movie S1 [file sciadv.abi7112_sm.pdf]

Science Advances
AAAS

Supplementary Materials for

Populations of local direction–selective cells encode global motion patterns generated by self-motion

Miriam Henning, Giordano Ramos-Traslosheros, Burak Gür, Marion Silies*

*Corresponding author. Email: msilies@uni-mainz.de

Published 19 January 2022, *Sci. Adv.* **8**, eabi7112 (2022)
DOI: 10.1126/sciadv.abi7112

The PDF file includes:

Figs. S1 to S6
Legend for movie S1

Other Supplementary Material for this manuscript includes the following:

Movie S1

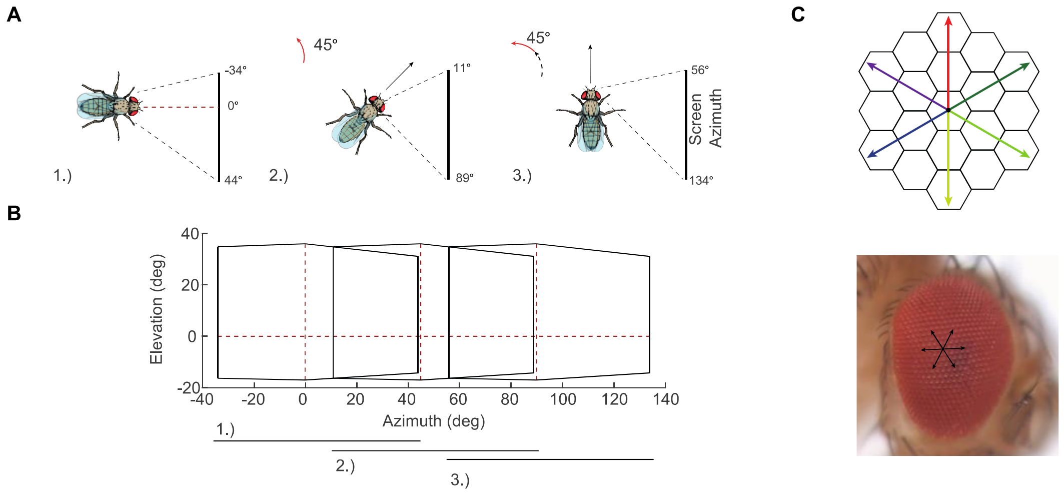

Fig. S1. Schematic of the experiment and the hexagonal arrangement of the fly eye. (**A**) Schematic of the experiment. The fly was positioned in three different orientations (0°, 45°, 90°) towards the screen to extend the visual field covered by one experiment, together spanning ~ 168° in azimuth. (**B**) Visual space stimulated by the screen is illustrated by solid lines for each rotation of the fly. Red dotted lines represent the plane with closest distance to the fly where the screen covered ~55° (-17° - 36°) in elevation. (**C**) The existence of six subtypes matches the number of diagonals in the hexagonal arrangement of the fly eye.

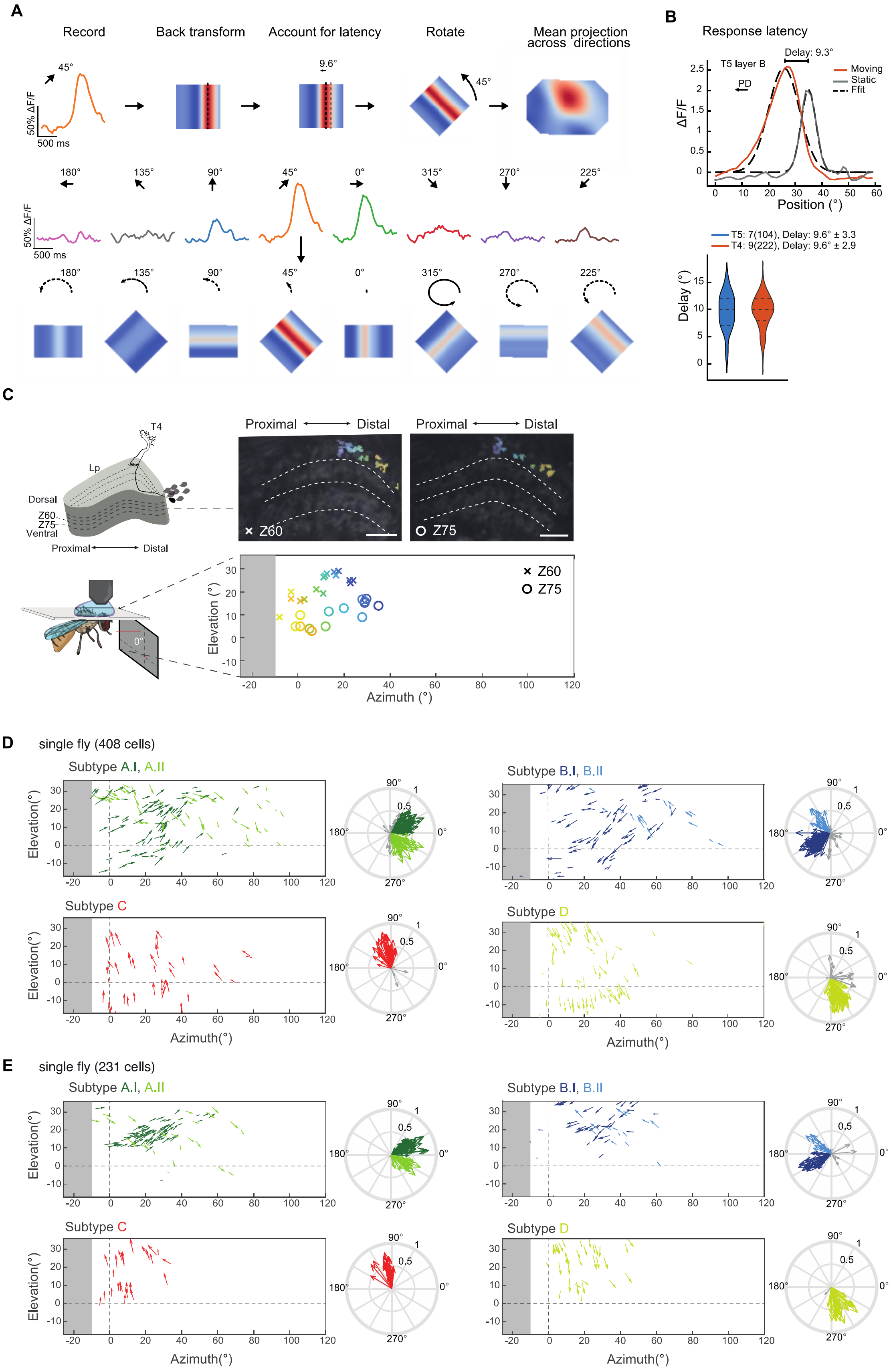

A
Record
Back transform
Account for latency
Rotate
Mean projection across directions
45°
9.6°
50% ΔF/F
500 ms
180°
135°
90°
45°
0°
315°
270°
225°
B
Response latency
T5 layer B
Delay: 9.3°
PD
Moving
Static
Ffit
ΔF/F
Position (°)
T5: 7(104), Delay: 9.6° ± 3.3
T4: 9(222), Delay: 9.6° ± 2.9
Delay (°)
C
T4
Lp
Dorsal
Z60
Z75
Ventral
Proximal
Distal
Z60
Z75
Elevation (°)
Azimuth (°)
D
single fly (408 cells)
Subtype A.I, A.II
Subtype B.I, B.II
Subtype C
Subtype D
Elevation(°)
Azimuth(°)
E
single fly (231 cells)
Subtype A.I, A.II
Subtype B.I, B.II
Subtype C
Subtype D
Elevation(°)
Azimuth(°)

Fig. S2. Average T4/T5 tuning changes gradually along the horizontal and vertical visual axis. (A) Illustration of the back transformation of T4/T5 responses to edges moving into eight different directions to map receptive-field centers (60). Response traces were transformed into two dimensional images and shifted by 9.6° to account for neuronal response latencies and calcium dynamics measured in T4/T5 cells (see (B) and methods). After rotating images according to the direction of the stimulus the mean projection across direction maps resulted in the receptive-field map. **(B)** Top: Response latency of T4/T5 neurons were measured by the spatial difference between responses to a static bar shown at different spatial locations (grey solid line) and a moving stimulus (orange solid line). Dotted lines depict the fits to the corresponding responses. Latency was 9.3° in this example. Bottom: The average delay measured for T4 and T5 neurons was 9.6°. Dotted lines represent the quartiles. **(C)** Mapping of the anatomical arrangement in the lobula plate and visual space. 2-photon image of the lobula plate showing example ROIs of T4/T5 axon terminals (top) and their corresponding receptive-field center positions in visual screen coordinates (bottom). Shown are receptive-field center positions plotted onto visual screen coordinates for one fly. Neighboring cells in anatomical space respond to neighboring points on the screen. The proximodistal anatomical axis roughly corresponds to the horizontal axis in visual space, whereas the dorsoventral anatomical axis roughly corresponds to the vertical axis on the screen (in these two examples, layer A only contain ROIs belonging to subtype A.I). **(D,E)** Individual T4/T5 tuning of all cells within one fly plotted at their receptive field center coordinates in visual space. Arrows depict tuning direction, vector length indicates direction selectivity. Each plot shows data from one anatomical layer, color-coded by class identity from the SNOB model. Horizontal and vertical dashed lines mark the split between left and right visual hemispheres and the horizon, respectively. Gray shaded areas indicate the visual space in the left hemisphere that cannot be seen by the right eye.

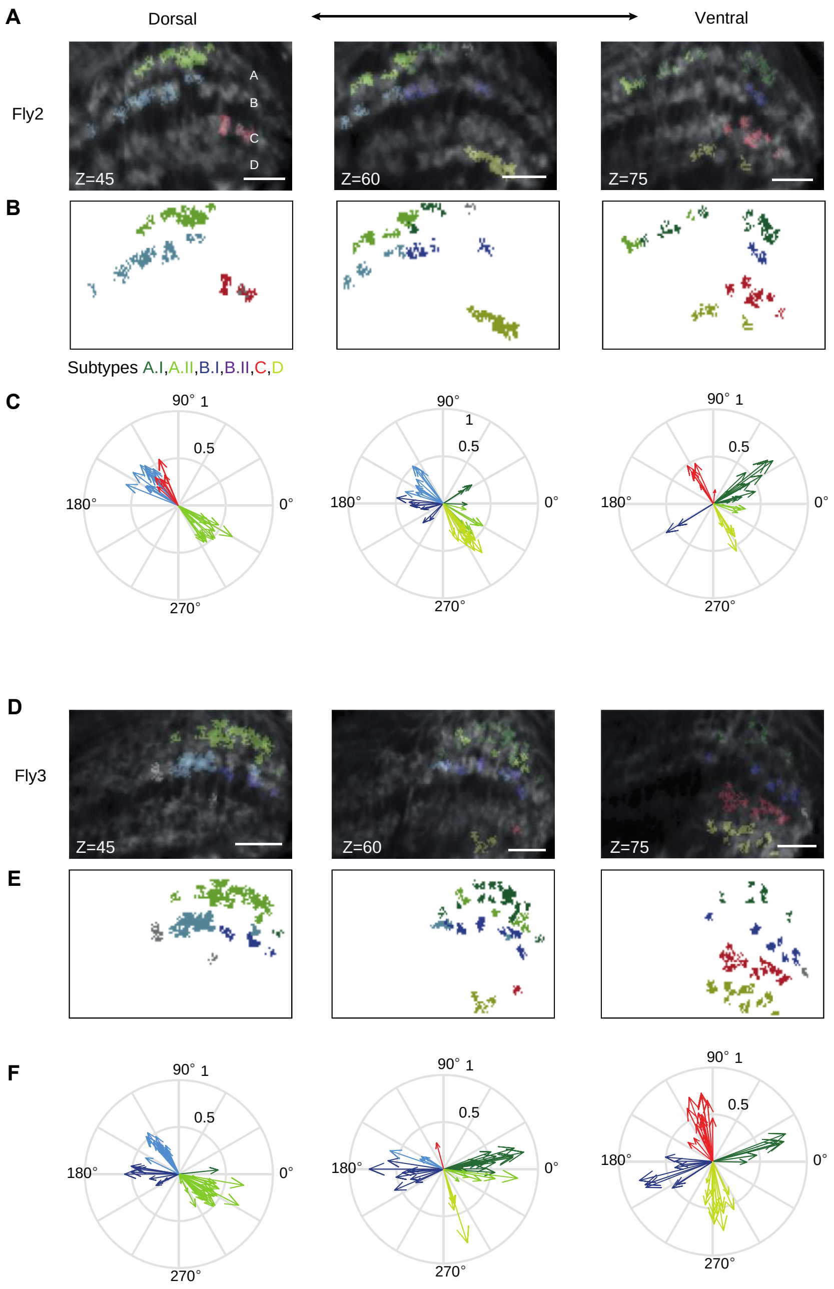

Fig. S3. Layer A and B subtypes are separated along the dorsoventral axis of the lobula plate. (A) 2-photon calcium images of the lobula plate at three different z-depths (Z), given in µm relative to T4/T5 cell bodies, recorded in one fly (see **fig. S1B**). ROIs are color-coded based on their subtype identity. **(B)** Same ROIs as in (A) shown without the 2-photon image for better visualization. **(C)** Compass plots showing directional tuning of the ROIs shown in the panels above. **(D-F)** Same as in (A-C) shown for another example fly. Individual images, corresponding to planes along the dorsoventral axis, predominantly show the two subtypes A.II and B.II, or the four subtypes A.I, B.II, C and D. Scale bar 10µm.

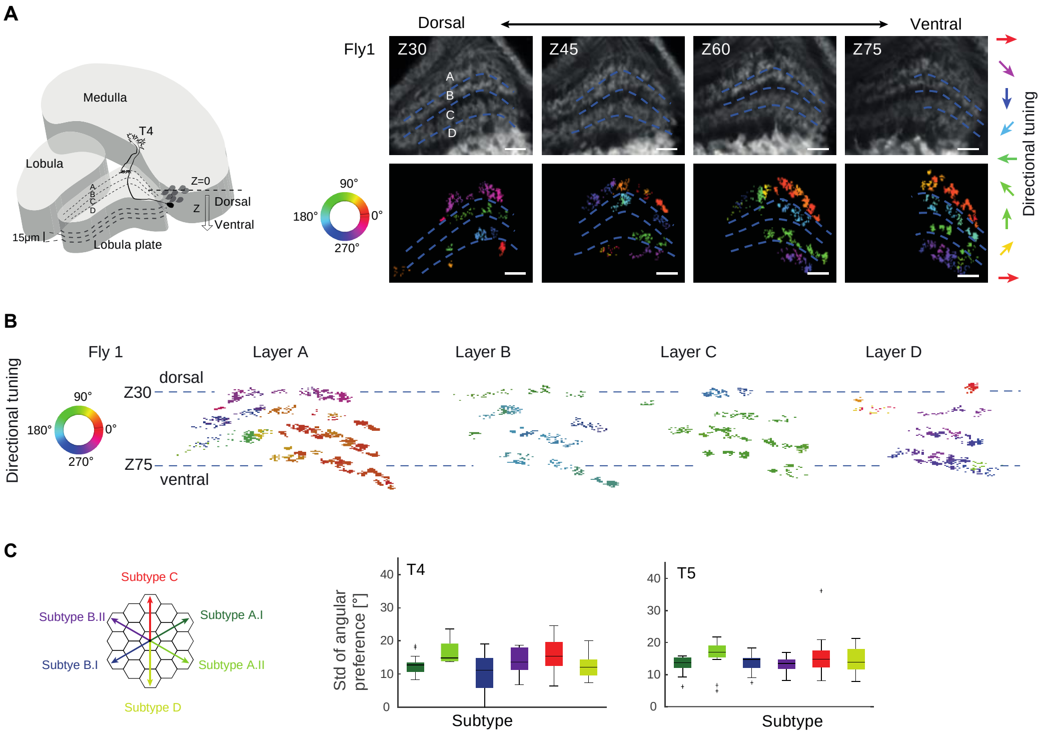

Fig. S4. Tuning within individual subtypes is topographically organized. **(A)** 3D illustration of the lobula plate. Recordings were made at different z-depths, corresponding to different locations along the dorsoventral axis. Example images from one fly recorded at different planes along the dorsoventral axis, shown as 2-photon images (upper row) or color-coded ROIs based on their tuning preference (lower row). Scale bar = 10µm. **(B)** Pseudo z-stacks of ROIs from each lobula plate layer recorded at different z-depths. Examples are shown from three different flies. Colors indicate tuning preference. The most prominent tuning difference was attributable to the two subtypes in layers A and B. Some more subtle changes in tuning were also apparent along the dorsoventral axis, but they were less prominent than along the proximodistal axis. **(C)** To analyze variability of T4/T5 directional tuning preference, the standard deviation of tuning for each of the six subgroups in each fly is plotted. Shown are mean ± standard deviations across flies (n=14). Tuning distribution between subtypes were not significantly different (Wilcoxon test, p>0.05).

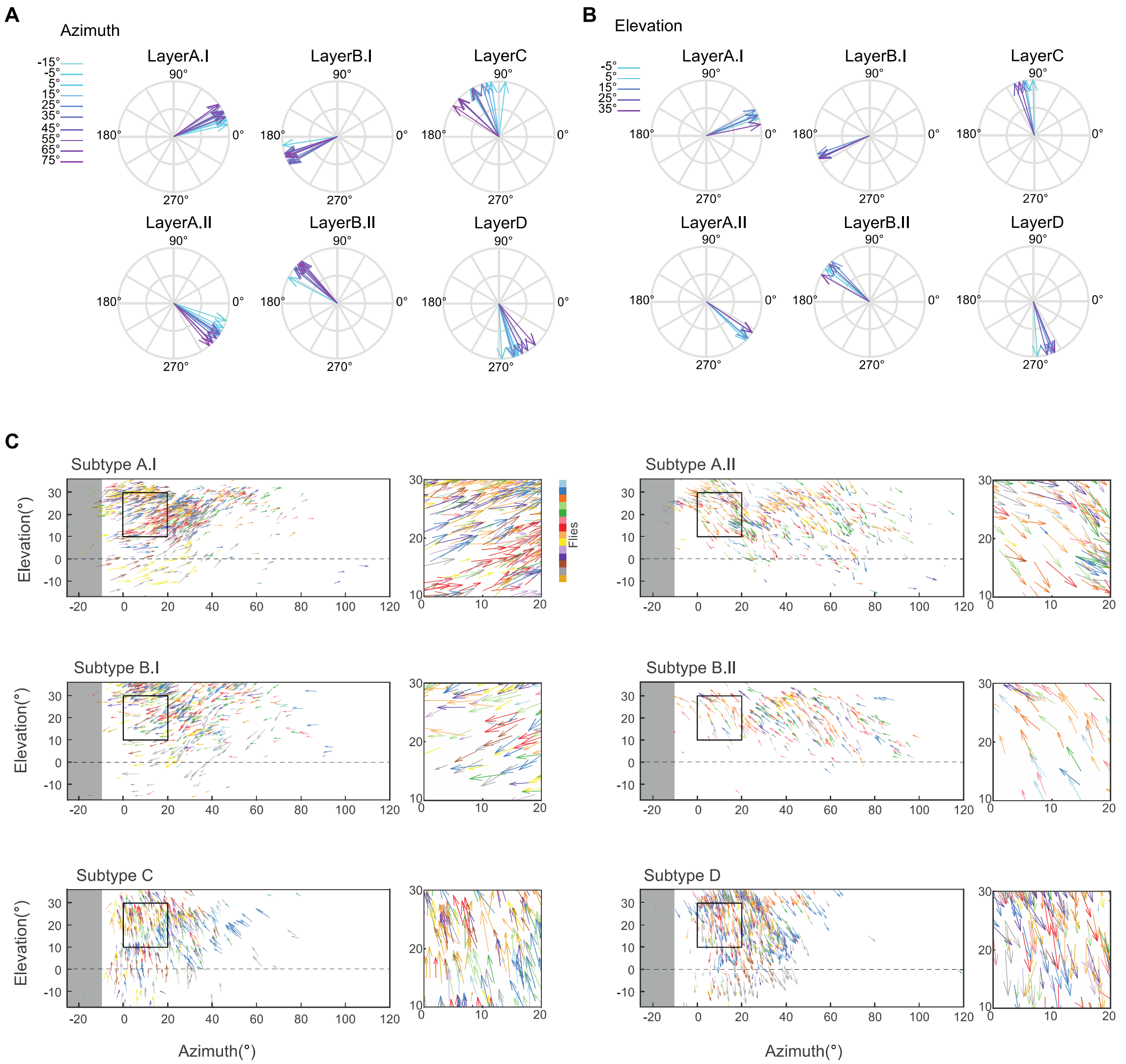

Fig. S5. T4/T5 tuning changes gradually, and global motion patterns are similar between flies. (**A, B**) Mean tuning direction from data recorded in 14 flies across 10° wide bins demonstrate that tuning shifts gradually along both the horizontal (A) and the vertical (B) dimension of the visual field. Same data as black arrows in Figure 5, plotted as compass plots. **(C)** Same data as in Figure 5, color-coded by fly identity, n=14 flies. Arrows indicate tuning direction of individual neurons plotted at their receptive field center coordinates in visual space. Length of vectors indicates direction selectivity of T4/T5 neurons. Horizontal and vertical dashed lines mark the split between left and right visual hemispheres and the horizon, respectively. Gray shaded areas indicate the visual space in the left hemisphere that cannot be seen by the right eye. The boxed area indicates the region magnified in the plot on the right side of each subtype panel. Note that vectors of different colors, indicating data from different flies, show similar tuning, indicating little fly-to-fly variability.

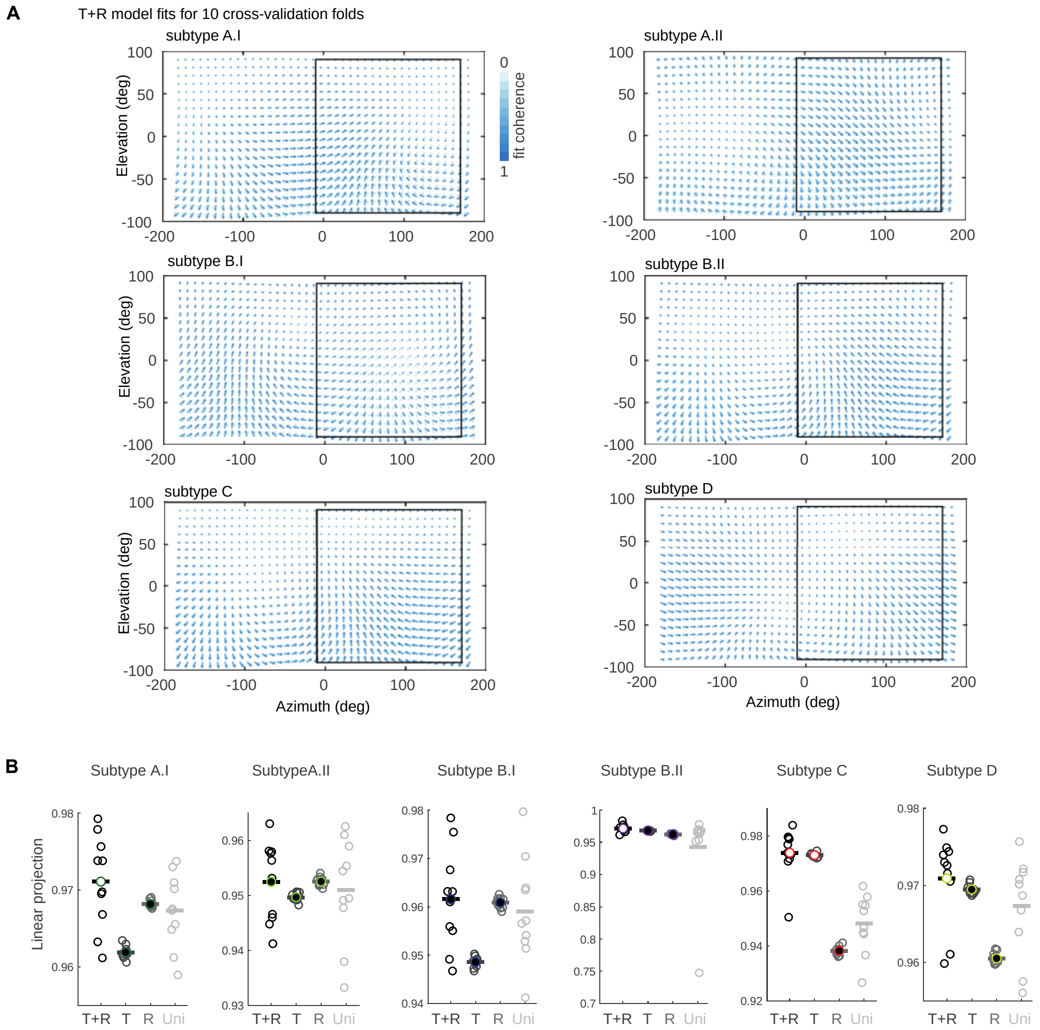

Fig. S6. Flow fields generated by self-motion are matched filters for T4/T5 population tuning. (A) Flow fields obtained from a model fitting both translation (T) and rotation (R) parameters. Arrows show results from ten cross-validation folds such that fit coherence between cross-validations is indicated by the opacity of vectors. For each subtype, a large region of visual space is shown, spanning 360º in azimuth and 200º in elevation. The black box indicates the extent of visual space that is seen by the right eye of the fly (48). **(B)** Quality of the fit obtained from linear projection of the model to the data, shown for four different model variations allowing to train either all parameters for translation and rotation (T+R), only translation (T), only rotation (R) or a model with uniform direction vectors (Uni). Each circle represents the best fit of 10-fold cross-validation (see methods). Colored circles represent the average of the ten cross-validation results. Open circles indicate a significant difference to the uniform model prediction (Wilcoxon: $p<0.001$)

Movie S1. Z-stack of T4/5 cells in the lobula plate. Two photon images of T4/T5 cells expressing GCaMP6f. In the movie the focus moves from dorsal to ventral in the lobula plate. Z-depth count was set to zero when the first cell bodies were encountered (Z 0) to ensure comparability between flies. Illustration pauses at the dorsal most (Z 30) and ventral most imaging planes where responses of cells were recorded. A,B,C,D indicates the four layers of the lobula plate.
